# Redox Modulation of Erythropoiesis by Selenoproteins

**DOI:** 10.1007/s12011-025-04923-4

**Published:** 2025-12-04

**Authors:** Hangdi Gong, Robert F. Paulson, K. Sandeep Prabhu

**Affiliations:** Department of Veterinary and Biomedical Sciences, Center for Molecular Immunology, and Center for Molecular Toxicology and Carcinogenesis, The Pennsylvania State University, University Park, PA 16802 USA

**Keywords:** Selenium, Anemia of inflammation, Stress erythropoiesis, Erythroblastic island, Glutathione peroxidase, SELENOW, SELENON, SELENOP, Macrophage polarization

## Abstract

Erythroblasts are constantly challenged by oxidative stress arising from the accumulation of free heme and reactive radicals, particularly during stress erythropoiesis, which is triggered by inflammatory signals. In response to infection or tissue damage, stress erythropoiesis serves as a compensatory mechanism to overcome inhibition of steady-state erythropoiesis by pro-inflammatory cytokines to sustain abundant red blood cell (RBC) production. Maturation of erythroblasts during stress erythropoiesis critically depends on the erythroblastic island (EBI) microenvironment, composed of a central macrophage and surrounding immature progenitors. Within this niche, the dynamic balance between inflammatory and resolution pathways is essential for proper erythroblast development and adaptation under stress. Selenium (Se), an essential trace element, incorporated into selenoproteins via the 21st amino acid selenocysteine, imparts antioxidant and redox-regulatory activity, which govern erythroblast-intrinsic signaling as well as microenvironmental regulation. Depletion of the selenoproteome or selenium in the diet results in mild anemia and impaired stress erythropoiesis. However, the contributions of individual selenoproteins to erythroblast development is not well understood. Given the importance of selenoproteins in influencing macrophage polarization, its contribution to the regulation of the erythropoietic microenvironment deserves further study. In this review, we highlight the role of selenium and selenoproteins in stress erythropoiesis, emphasizing their functions in supporting erythroblast development and modulating the erythropoietic niche.

## Selenium in Erythropoiesis and Anemia

Selenium is an essential trace element for human and animal health that functions as a component of 25 different selenoproteins (24 in mice) as selenocysteine (Sec), the 21st amino acid decoded by a penultimate UGA codon [1]. According to their structural homology and function, selenoproteins are classified into glutathione peroxidases (GPXs), thyroid hormone deiodinases (DIOs), thioredoxin reductases (TXNRDs or TRs), and other selenoproteins [1]. Selenoproteins are well-known for their role in redox homeostasis, underscoring their critical function in erythroblast development, especially during inflammation-induced stress erythropoiesis. Serum selenium is positively correlated with hemoglobin and serum iron levels in elderly population, where low serum selenium leads to higher risk of anemia [2, 3]. Selenium deficiency is commonly observed in primary anemias, such as thalassemia major (TM) [4] and sickle cell disease (SCD) [5], as well as in systemic diseases, where anemia frequently arises as a secondary complication, including chronic kidney disease (CKD) [6], cardiovascular disease (CVD) [7, 8], heart failure [9], and myelodysplastic syndrome [10]. These clinical studies indicate a potential protective role of selenium in supporting erythropoiesis during anemia-associated conditions. Experimental rodent models have yielded significant mechanistic data confirming the role of selenium in erythropoiesis. Mice maintained on selenium deficient (Se-D) diet were mildly anemic, along with increased oxidative stress indicated by higher levels of forkhead transcription factor (FoxO3a) and hypoxia-inducible factor-(HIF)1a [11]. Notably, selenium is indispensable for erythroblast development during stress erythropoiesis, which compensates for the decrease in erythroid output caused by inhibition of steady state erythropoiesis by pro-inflammatory cytokines [12]. In Se-D mice, phenylhydrazine (PHZ)-induced hemolytic anemia was lethal, characterized by impaired maturation of proerythroblast (ProE) to basophilic erythroblast (BasoE), together with the defective recruitment and development of spleen macrophage/monocyte microenvironment [13]. Studies have shown that dietary selenium supplementation as seleno-L-methionine (Se-Met, organic form) or sodium selenite (Na_2_SeO_3_, inorganic form) facilitate stress erythropoiesis through impacting distinct cell types in PHZ-induced hemolytic anemia model [14]. While Se-Met supplementation promoted stress erythroid progenitor development, Na_2_SeO_3_ facilitated the maturation of recruited monocytes supporting the erythropoietic microenvironment for stress erythropoiesis [14]. In response to the lack of selenoproteins, as in mice lacking the gene tRNA^[Sec]^ (*Trsp*) in macrophage, the transcription factor nuclear factor erythroid 2-related factor 2 (Nrf2) was upregulated to compensate and counter the redox stress [15]. Administration of polyinosinic: polycytidylic acid (poly (I: C)) to mimic viral infection and trigger toll-like receptor 3 (TLR3)-dependent innate immune response revealed that *Trsp* depletion led to mild anemia along with reduction in B lymphocytes [16]. This effect was exacerbated by lack of *Nrf2*, indicating the importance of redox homeostasis in erythropoiesis, where Nrf2 and selenoproteins may play a synergistic role [17]. Collectively, these studies demonstrate an important role for selenoproteins in erythroblast development. The distinct functions of individual selenoproteins in erythropoiesis remain unclear, which offers a new frontier of study in erythropoiesis. Here we review the literature in an effort to provide new ideas for the various selenoproteins, abundantly expressed in cells that are key for erythroid development.

## Oxidative Damage to Erythroblast and Erythropoiesis

Red blood cells (RBCs) are constantly exposed to endogenous reactive oxygen species (ROS) and reactive nitrogen species (RNS). ROS, including superoxide anion (O_2_^⋅−^), hydrogen peroxide (H_2_O_2_), and hydroxyl radicals (OH^⋅^), are the byproducts and sequential products during hemoglobin autoxidation in RBCs [18]. Hemoglobin autoxidation is a spontaneous process where the oxyhemoglobin (HbO_2_) is converted to methemoglobin (MetHb) and superoxide, while ferrous heme iron (Fe^2+^) is oxidized to ferric (Fe^3+^) form [19]. RNS, such as nitric oxide (NO⋅) and peroxynitrite (ONOO^−^), also contribute to oxidative stress in RBCs. Nitric oxide converts HbO_2_ into MetHb, which impairs oxygen transport, as MetHb lacks the ability to reversibly bind oxygen [20]. Unstable superoxide undergoes dismutation to H_2_O_2_ that undergoes Fenton reaction by heme and iron to produce the reactive hydroxyl radical, which oxidize biomolecules leading to RBC membrane damage [21]. During the formation of functional hemoglobin (Hb), the coordination of heme insertion into globin chains also contributes to oxidant production. Hemoglobin A (HbA), the most common form of adult hemoglobin, is composed of two α and β globin subunits [22]. Excessive unpaired globin chains, especially structurally unstable α-globin, produce superoxide through autoxidation, and their degradation releases free heme and iron that drive Fenton reaction, leading to hemoglobinopathies and anemia [23, 24]. During inflammation, hemolytic disorders, or selenium deficiency, the imbalance of ROS/RNS leads to excessive oxidative stress, which impairs erythroblast development, ultimately contributing to anemia and impaired oxygen delivery [21, 25, 26]. Excessive ROS can trigger lipid peroxidation and membrane damage, leading to premature cell death. Oxidation of hemoglobin also promotes formation of Heinz bodies composed of denatured hemoglobin, marking erythroblasts for clearance and thereby limiting red cell output [27]. Under Se-D conditions, accelerated oxidative stress enhances hemoglobin denaturation and aggregation, leading to the formation of increased Heinz bodies within erythrocytes [11].

Erythroblasts and mature RBCs rely heavily on antioxidant defense systems against ROS/RNS-induced damage, including superoxide dismutase (SOD)1 and SOD2, which convert superoxide to H_2_O_2_ that is a substrate for catalase, selenium-dependent GPXs [28], and non-selenium dependent glutathione S-transferases (GSTs) with peroxidase activity, where it is reduced to H_2_O [29]. In addition to H_2_O_2_, GPXs also reduce lipid hydroperoxides using glutathione (GSH) as an electron donor, while thioredoxin reductases (TXNRD1, TXNRD2, and TXNRD3) also display additional disulfide oxidoreductase activity to maintain redox homeostasis, and peroxiredoxins (PRDXs) further assist in efficiently reducing peroxides in cells [30–34]. ROS-induced DNA damage activates p53-dependent checkpoints, causing cell-cycle arrest or apoptosis that further reduces progenitor expansion [35]. Dysregulated iron metabolism, through Fenton-driven reactions and ferroptotic pathways, amplifies oxidative injury and disrupts hemoglobin synthesis [36]. These mechanisms are crucial for preventing oxidative damage to the membrane, hemoglobin oxidation, and impaired erythroblast development.

## Stress Erythropoiesis and Inflammation

Steady state erythropoiesis generates >10^11^ erythrocytes per day in the bone marrow of a healthy individual to replace senescent erythrocytes removed by the spleen and liver [37]. There is a tight relationship between oxygen carrying capacity and blood viscosity, which maximizes oxygen delivery while minimizing blood viscosity. Decreases in oxygen delivery can be compensated for by increasing erythropoietin (Epo) production, which promotes the differentiation of late-stage erythroid progenitors, increasing erythrocytes in the periphery. However, infections or tissue damage that cause inflammation reduce steady-state erythroid output by inhibiting erythroid differentiation. Increasing Epo levels is ineffective as pro-inflammatory cytokines inhibit Epo- dependent differentiation. To compensate for this decrease in production, stress erythropoiesis is initiated. Stress erythropoiesis employs a different strategy from the constant production seen in steady-state erythropoiesis and more closely resembles tissue regeneration processes observed in muscle and lung following injury. In mice, short-term hematopoietic stem cells (ST-HSCs) migrate along with monocytes to the spleen where they develop into stress erythroid progenitors (SEPs) and the splenic stress erythropoiesis niche, respectively. In humans, the role of the spleen as the site of stress erythropoiesis has not been definitively established, but ST-HSCs and monocytes are mobilized into the peripheral blood during inflammatory anemia to generate SEPs that respond to same signals and express the same markers as murine SEPs. The initial step in this process is to expand a population of transient amplifying cells that are restricted to the erythroid lineage and self-renewing. This expansion is driven by developmental signals like bone morphogenetic protein (BMP4), Indian hedgehog (IHH) and canonical Wnts, which act in concert with same pro-inflammatory cytokines that inhibit steady state erythropoiesis. These SEPs then transition to erythroid committed progenitors, which lose their ability to self-renew and become stress burst-forming unit-erythroid (BFU-E). This transition is driven by pro-resolving signals like Epo, prostaglandin J_2_ (PGJ_2_) and prostaglandin E_2_ (PGE_2_). The terminal differentiation of stress BFU-E and colony-forming unit-erythroid (CFU-E) cells is similar to steady-state erythropoiesis. Erythroblast maturation begins with ProE, which are characterized by enlarged nuclei and a larger cell size. As they divide and differentiate, they become BasoE and polychromatic erythroblasts (PolyE) with robust hemoglobin and protein synthesis. These cells further differentiate into the orthochromatic erythroblast (OrthoE), marked by condensed nuclei and accumulated hemoglobin. Upon enucleation, erythroid precursors mature into reticulocytes containing residual RNA. Finally, after the clearance of residual rRNA, mature erythrocytes enter the circulation [38, 39]. Mice fed a Se-D diet developed mild anemia and showed impaired stress erythropoiesis during recovery from hemolytic anemia. Se deficiency significantly delayed the migration and expansion of SEPs and further impaired the maturation from ProE to BasoE, resulting in delayed and insufficient recovery of erythropoiesis [11, 13].

During stress erythropoiesis, given the excessive demand for heme, iron, and cytokines, erythroblast development occurs within specialized structures known as erythroblastic islands (EBIs). Within these EBIs, central macrophages assume a crucial role as nurturing or “nursing” the surrounding erythroblasts, facilitating the proliferation and differentiation of SEPs into fully mature RBCs [26].

Systemic inflammation serves as a critical signal that triggers stress erythropoiesis [40]. Anemia of inflammation (AI) or also called anemia of chronic disease (ACD) is the second most common anemia following iron deficiency anemia (IDA) [41]. Pro-inflammatory signals, including interleukin-6 (IL-6) [42, 43], IL-1β [44], interferon gamma (IFNγ) [45] and tumor necrosis factor-alpha (TNFα) [46], skew hematopoietic stem cell (HSC) fate towards myeloid effector cells to counter the infection, which inhibits steady-state erythropoiesis [26]. IL-6 and IL-1β stimulate expression of hepcidin, the iron-regulatory hormone, causing degradation of ferroportin and iron retention in macrophages, which elevates labile Fe²⁺ and lipid peroxidation [47, 48]. This pro-ferroptotic pressure is countered by selenoprotein-dependent antioxidant defenses by selenoenzyme GPX4 [49]. Studies have shown that Ly6C^hi^ monocyte-derived macrophages and liver-resident macrophages, instead of red pulp-macrophages, play a major role of iron recycling as well as erythrophagocytosis [50], supporting stress erythropoiesis in the spleen. The hypoxic and iron-deficient inflammatory environment impairs Epo-induced JAK/STAT signaling, leading to insufficient RBC production [51, 52]. During this period, transiently amplifying SEPs, which do not produce heme or hemoglobin, allow stress erythropoiesis to continue under adverse conditions. GPXs and TXNRDs alter inflammatory signaling and oxidative stress in macrophages, indirectly regulating iron availability and stress erythropoiesis.

Despite the negative effects of inflammation on steady-state erythropoiesis, pro-inflammatory signals stimulate erythrophagocytosis in the spleen, where heme that is released signals to enhance the expression of the Ets-family transcription factor Spi-C [40]. Spi-C mediates the development of monocytes into macrophages in the niche and drives the expression of growth/differentiation factor 15 (GDF15), a protein hormone in the TGFβ family released by stressed and/or inflamed cells, and increases BMP4 in the niche [53]. Although pro-inflammatory signals increase the expression of hepcidin to restrict iron availability, the expansion of SEPs driven by increased Epo production from the kidney induces the expression of hepcidin antagonist, erythroferrone (Erfe), facilitating iron release for continued stress erythroblast maturation [54].

A recent study revealed that the commitment of SEPs to differentiation during stress erythropoiesis is intriguingly regulated by an Epo/Stat5-dependent signaling that involves macrophage-derived PGJ_2_ production [55]. PGJ_2_ is an arachidonic acid-derived cyclopentenone prostaglandin (PG) metabolite that functions as an anti-inflammatory bioactive lipid mediator and an endogenous ligand for PPARγ [56]. Wnt signaling that promotes SEP proliferation is inhibited by Epo/Stat5 signaling, thereby skewing SEP from proliferation towards differentiation. Elevated PGJ_2_ activates PPARγ, which suppresses Wnt expression in macrophages [57]. Interestingly, Epo/Stat5 signaling also promotes the production of PGE₂, suggesting that multiple PGs derived from arachidonic acid may act in parallel to coordinately regulate stress erythropoiesis under inflammatory conditions.

Prostaglandin J_2_, as well as its derivative 15-deoxy-Δ¹²,¹⁴-prostaglandin J₂ (15d-PGJ₂), are well known for their anti-inflammatory effect through binding to PPARγ, NF-κB, Nrf2, AP-1, and other proteins [58–61]. Recent study from our laboratory further demonstrated that itaconate, a pro-resolving metabolite of *cis*-aconitate, promoted SEP differentiation through activation of the Nrf2 activity [62]. Notably, a tightly regulated balance between pro-inflammatory and pro-resolving signals is essential not only for stress erythropoiesis, but also for steady-state erythropoiesis. Polarization of EBI macrophages from a pro-resolving (alternatively-activated ) to a pro-inflammatory (classically-activated) phenotype has been shown to impair erythropoiesis upon stimulation with GM-CSF [63] and in sickle cell patients [64]. Thus, the regulation of redox homeostasis through modulation of inflammation and resolution responses within the EBI environment becomes crucial for effective stress erythropoiesis.

Selenoproteins have been recently known for their role as antioxidant gate keepers that attenuate pro-inflammatory program, including cyclooxygenase-2 (COX-2), TNF-α, iNOS, IL-1β, and IL-12 [65, 66] and initiate pro-resolution programs to facilitate resolution [67]. Notably, during inflammation, selenium supplementation in macrophages is reported to skew arachidonic acid metabolism towards increased PGJ_2_ and decreased PGE_2_ production, highlighting a regulatory role in eicosanoid balance, while also indicating the potential of selenoproteins to serve as modulators of inflammation-induced erythropoietic dysfunction through redox-dependent mechanisms.

## Selenoproteins in Erythropoietic Microenvironment

Selenoproteins have been identified as a critical regulator in modulating HSC cell fate. Conditional HSC-specific selenoprotein depletion (with *Trsp*^fl/fl^*Mx1*^Cre^) in mice impaired HSC self-renewal, disrupted B cell maturation, and skewed HSCs towards myeloid lineage [16]. In response, a compensatory Nrf2 signaling mechanism was activated. Moreover, vitamin E, a ferroptosis inhibitor, could partially alleviate the B-cell maturation impairment, underscoring the importance of GPX4 in HSC cell fate regulation [16]. The *Trsp*^fl/fl^*Mx1*^Cre^ mice also developed macrocytic anemia, indicated by reduced hemoglobin level, elevated mean corpuscular volume (MCV), erythroblast degradation in peripheral blood, and splenomegaly due to induced stress erythropoiesis [16]. These findings further corroborate previous studies that demonstrate deficiency of Se in mice severely affects stress erythropoiesis, thereby exacerbating anemia.

A previous study from our lab has shown that mice on a Se-D diet exhibited delayed monocyte recruitment and impaired maturation into red pulp macrophages compared to mice on a selenium-adequate (Se-A) diet. This delay compromises EBI formation, further impairing stress erythropoiesis [13]. Consistently, *Trsp*^fl/fl^*LysM*^Cre/−^ mice exhibited delayed recovery from hemolytic anemia due to defective Spi-C-mediated monocyte recruitment and RPM maturation [13]. Interestingly, transplantation of Se-A supplemented monocytes into Se-D mice partially rescued this defect, highlighting the cooperative role of selenoproteins derived from both macrophages and erythroblast precursors in regulating stress erythropoiesis [13].

As well-known antioxidants, selenoproteins attenuate pro-inflammatory signals in macrophages [65]. Selenite treatment of bone marrow-derived macrophages reduced COX-2 and TNFα expression, indicating an anti-inflammatory effect [65]. COX-2 is an enzyme induced by inflammatory stimuli that catalyzes the conversion of arachidonic acid into bioactive lipid mediators, including PGE₂, PGD₂, and PGJ₂, influencing the balance between pro-inflammatory and anti-inflammatory signaling [58]. Under NF-κB-mediated stimulation by LPS, selenoproteins upregulate hematopoietic prostaglandin D₂ synthase (H-PGDS) expression in macrophages via a PPARγ-dependent positive feedback loop, enhancing the production of anti-inflammatory cyclopentenone PGs, such as PGJ₂ and 15d-PGJ₂. Elevated PGJ₂ and 15d‑PGJ₂ subsequently inhibit NF-κB signaling, leading to reduced PGE_2_ production through suppression of microsomal prostaglandin E synthase-1 (mPGES-1) and thromboxane synthase (TXS) [68]. Collectively, these studies demonstrate that selenoprotein supplementation can modulate macrophage polarization from a classically activated, pro-inflammatory state toward an alternatively activated, pro-resolving phenotype. Together with Epo/Stat5-driven PGJ_2_ production, these findings suggest that selenoproteins regulate stress erythropoiesis by modulating macrophage polarization in EBI macrophages. Furthermore, in the *Citrobacter rodentium* (a Gram -ve bacterium) infection model, depletion of selenoprotein in either macrophages or neutrophils led to poor resolution of inflammation [69, 70]. Deletion of selenoproteome in the neutrophilic compartment reduced neutrophil apoptosis followed by impaired efferocytosis by macrophages, causing severe tissue damage [69]. 

## Selenoproteins in Erythroid Cells and Erythropoiesis

### Glutathione Peroxidase (GPX)

These are the most well-studied family of selenoproteins that protect RBCs from oxidative stress by reducing H_2_O_2_ and lipid hydroperoxides. GPX1 is the most abundant GPX isoform in RBCs [71], and is highly expressed during erythropoiesis. It was the first purified selenoprotein, originally isolated from RBCs [72]. The GPXs efficiently degrade H_2_O_2_ to H_2_O, while oxidizing GSH to oxidized glutathione (GSSG). GSSG is then reduced by glutathione reductase (GR) that is dependent on nicotinamide adenine dinucleotide (NADPH). As a key antioxidant defense against oxidative stress in erythroblast, GPX1 especially protects RBC from high level endogenous and exogenous peroxides [73], indicating its critical role under abnormal stress conditions, such as inflammatory stress, to protect the lipid membrane [74]. Interestingly, *Gpx1*^*−/−*^ mice do not show a defect in peroxide protection in RBC, indicating compensatory mechanisms in erythroblasts, which include enzymes such as thiol-dependent peroxiredoxins (PRXs) [74]. PRX2 serves as a barrier against endogenous H_2_O_2_ and other peroxides to corresponding alcohols [45] and is also known to synergistically couple with GPX1 [75]. However, the oxidation state of PRX2 is a critical determinant of its activity, thereby highlighting the complementary role of selenoproteins in erythroblast redox protection, helping limit PRX2 overoxidation and preserve its activity [76].

GPX4 has been identified as a key GPX isoform critical in hematopoiesis and erythropoiesis [77]. GPX4 is expressed in most cell types and tissues, albeit at lower levels compared to GPX1 in RBC [78]. GPX4 is a phospholipid hydroperoxide glutathione peroxidase that catalyzes the reduction of phospholipid hydroperoxides (PLOOHs) to non-toxic phospholipid alcohols (PLOHs) using GSH as a cofactor [1, 79]. GPX1, GPX2, and GPX3 primarily reduce soluble hydroperoxides, while GPX4 is uniquely adapted to reduce PLOOHs within biological membranes. GPX4 is an essential regulator of ferroptosis, a highly regulated form of cell death characterized by iron-dependent lipid peroxidation, independent from other forms of cell death, such as apoptosis and necroptosis [49]. Abnormal erythropoiesis, such as in β-thalassemia, is characterized by erythroblasts with heightened ROS, most evident in the later phases of erythroid maturation [80]. Disrupted iron overload cause abundant influx of Fe^3+^ through transferrin receptor (TfR1; CD71), leading to accumulation Fe^2+^ iron via six-transmembrane epithelial antigen of prostate 3 (STEAP3), which can fuel heme biosynthesis but also exacerbate ROS production through Fenton reaction [80, 81]. The abundant heme iron accelerates ROS production, causing imbalance in the cellular oxidative homeostasis. Heat shock protein b-1 (HSPB1) modulates iron uptake, in part, by limiting the availability of TfR1 [82], while Nrf2 regulates pathways involved in the degradation of heme, thereby comprising two key intertwined regulatory mechanisms of ferroptosis [83]. *Mx1*^Cre^ -driven conditional deletion of *Trsp* primarily in hematopoietic and immune cells led to Nrf2-dependent compensation of ROS scavenging in erythroid cells highlighting a coordination between Nrf2 and selenoproteins, especially GPX4 [17].

Retrospective analyses of existing RBC proteomics and metabolomics studies revealed enzymatic activity of GPX4 correlated with lipid-anchored proteins [84]. GPX4 depletion in HSCs caused systemic hepatic iron overload, which impaired erythroblast development and stress erythropoiesis [85]. Operating in parallel with GPX4, vitamin E (α-tocopherol) modulated lipid peroxidation by scavenging lipid radicals. Thus, vitamin E deficiency further aggravated oxidative damage leading to higher HSC death [77]. Interestingly, GPX4 depletion in mouse erythroid precursors induced receptor-interacting protein kinase 3 (RIP3)-dependent necroptosis, but not ferroptosis, which could be rescued by vitamin E supplementation [86]. GPX4 is suggested to be critical in reticulocyte enucleation through a non-ferroptosis process. While lipid peroxides initiate ferroptosis, these lipid peroxides can also form reactive aldehyde intermediates such as malondialdehyde (MDA) and 4-hydroxy-2-nonenal (4-HNE) that can deplete cellular GSH as well as covalently modify protein thiols thereby exacerbating redox stress [80]. GPX4 depletion in HSCs impairs reticulocyte enucleation due to increased accumulation of lipid peroxides, which inhibits mitochondrial lysis [85]. RSL3-induced irreversible inhibition of GPX4 leads to lipid raft clustering to reduce phosphorylation of myosin light chain (MLC) in reticulocytes, which is independent of ferroptosis, necroptosis, apoptosis, and mitophagy, impair reticulocyte enucleation [87]. Together, these findings highlight GPX4 as a multifunctional regulator in hematopoiesis, erythropoiesis, and reticulocyte maturation, where its role extends beyond ferroptosis in maintaining lipid homeostasis and cellular integrity.

### Selenoprotein P, Selenoprotein W, Selenoprotein N, and Other Selenoproteins

In addition to GPXs, functions of other selenoproteins as antioxidants also potentially facilitate erythropoiesis, including stress erythropoiesis under anemic and/or inflammatory conditions. However, much less is known about these proteins. In the sections below, we highlight a few selenoproteins and their plausible role in erythropoiesis.

Selenoprotein P (SELENOP) is a plasma protein containing multiple selenocysteine (Sec) residues and primarily secreted from liver, although extrahepatic expression in specific cell types is also known [88]. SELENOP mainly functions as a transporter of selenium impacting different organs and tissues, including brain, testis, heart and kidney [89], where Sec is used for incorporation into other selenoproteins for redox regulation and other cellular functions. Studies have shown that low SELENOP in acute heart failure patients was associated with lower hemoglobin and increased TfR1, which impairs erythropoiesis to promote anemia [90]. In thalassemia major, lifelong transfusions cause iron overload with thyroid dysfunction, and circulating SELENOP is correspondingly higher and inversely correlated with thyrotropin (TSH) [91]. While the exact role of SELENOP is not known in erythroblast development, given its role as the selenium transporter throughout the body, and the fact that the expression of SELENOP correlates well with total selenium levels [92], it is plausible that the expression of other selenoproteins in erythroblast development respond to the levels of SELENOP. Although transcriptomic data suggest low-to-moderate expression of apolipoprotein E receptor 2 (ApoER2, LRP8), one of the receptors for SELENOP, on HSCs [93], its role as a SELENOP receptor or an ApoE receptor in hematopoiesis remains unclear. Further studies in this area might shed light on the temporal control of selenoprotein expression during erythroblast development and whether selenium incorporation is prioritized towards specific selenoproteins suggesting a hierarchy.

Another critical selenoprotein that regulates stress erythropoiesis is selenoprotein W (SELENOW). SELENOW is a 9-kDa GSH-dependent selenoprotein containing a thioredoxin-like fold with a conserved CxxU (C: Cys; U: Sec) motif that functions as disulfide reductase. SELENOW expression is highly responsive to dietary selenium [14]. Compared to the Se-D diet, SELENOW was among the most significantly upregulated proteins in mice maintained on a Se-A diet, especially in ProE and BasoE, highlighting its potential role in erythroblast maturation during stress erythropoiesis [13]. Depletion of SELENOW suppressed the in vitro growth of stress BFU-E cell colonies and induced defects in terminal maturation of murine erythroblast cell line, G1E. In the GATA1-null G1E cell line, ER4, SELENOW depletion significantly reduced G2/M phase cell [13]. SELENOW facilitated G2/M transition after DNA damage through dissociating 14-3-3 from CDC25B [94]. SELENOW depletion in epithelial cells also induced G1 phase arrest [95], indicating a potential role of SELENOW in regulating cell cycle during erythroblast maturation.

Apart from the selenoproteins described above, the identities of other selenoproteins that potentially regulate erythroblast development remain to be characterized. Based on a previous study, selenoprotein N (SELENON) is another candidate selenoprotein that may have a role in stress erythropoiesis. Complete loss of the ER-resident protein SELENON caused a significantly greater impairment in the recovery from PHZ-induced acute hemolytic anemia than in heterozygous mice, as indicated by lower hematocrit (Hct) [96]. According to its function in maintaining intracellular calcium homeostasis in muscle satellite cells through the modulation of sarcoendoplasmic reticulum (SR) calcium transport ATPase (SERCA) activity, SELENON could serve as a potential regulator during the differentiation of SEP, which show similar characteristics to muscle satellite cells [96]. Another noticeable protein that may regulate erythropoiesis is selenium-binding protein 1 (SBP1; SELENBP1). It has been characterized as a conserved copper-dependent thiol oxidase that binds selenium but does not contain Sec amino acid residue [97, 98]. SBP1 protein was upregulated in human bone-marrow differentiated CD34^+^ progenitor cells that was stimulated with Epo [99]. Given its role in binding selenium and redox-dependent regulation of cell proliferation [80, 81], the crosstalk of SBP1 with the selenium availability for selenoprotein synthesis could be an important pathway in erythroblast development highlighting the interaction between selenium, copper, and iron.

**Fig. 1:**
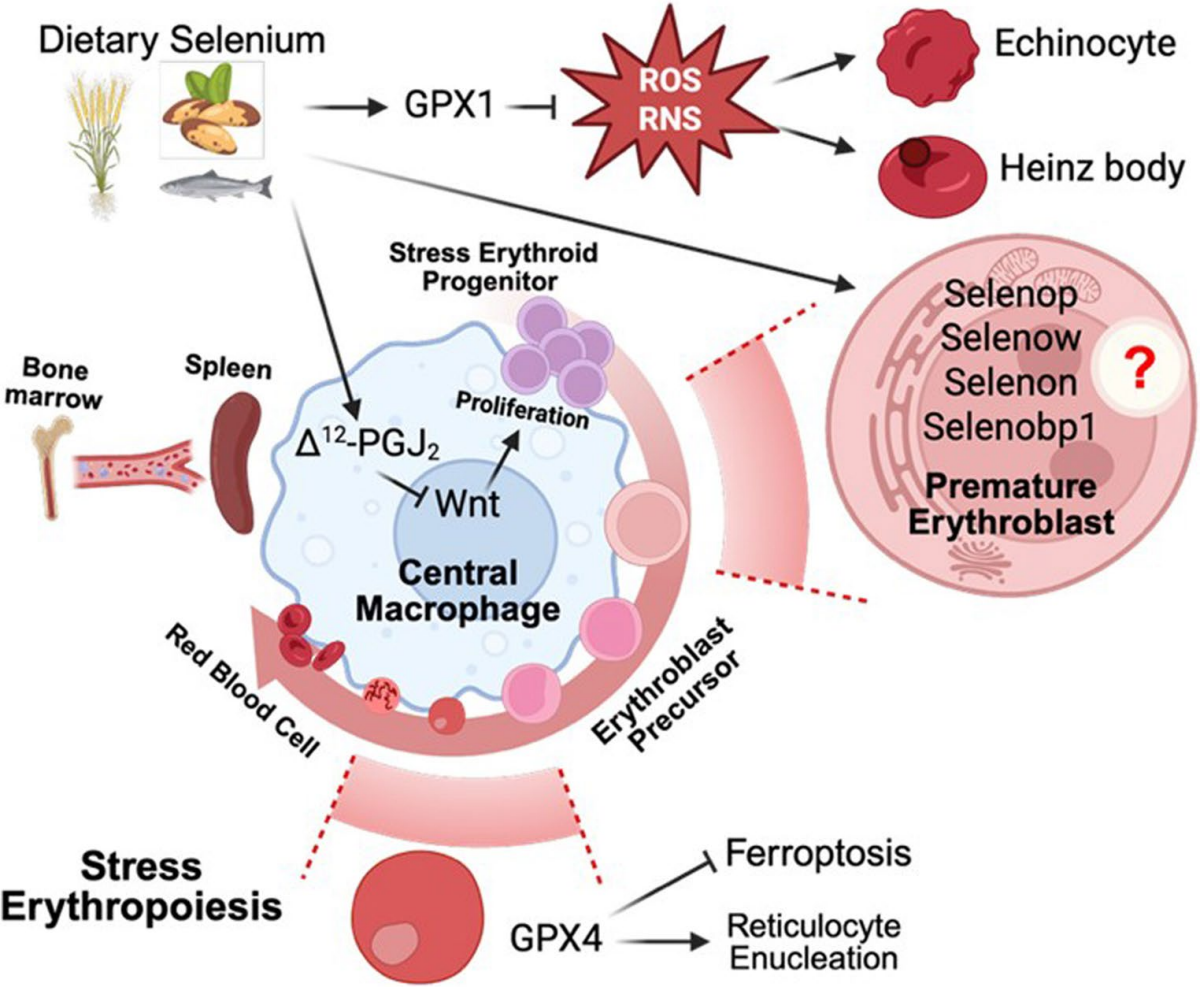
Selenium-mediated regulation of erythropoiesis and stress erythropoiesis. Dietary selenium supports erythroblast development and stress erythropoiesis during anemia recovery. GPX1 attenuates redox-enriched environment in erythrocyte and erythroblast precursors to prevent ROS-induced cytotoxicity. GPX4, as the main regulator of ferroptosis, cooperates with Vitamin E to regulate reticulocyte maturation. In stress erythropoiesis, selenoproteins in EBI macrophages promote a pro-resolving environment by inducing COX1-mediated PGJ₂ production, which potentially inhibits Wnt signaling to shift SEPs from proliferation to differentiation. The contributions of other selenoproteins to erythroblast precursor development remain an open and intriguing question. Schematic generated using Biorender.com

As shown in Fig. 1, in murine models, selenium-deficiency leads to mild anemia that worsens following hemolytic stress, highlighting the critical role of selenoproteins in regulating erythropoiesis through redox-dependent mechanisms. Lack of selenoproteome skews the fate of HSCs toward myeloid lineage, with reduced B-cell output and increased splenic erythropoiesis, underscoring the profound impact of selenoproteins on the HSC niche. In macrophages, selenoproteins attenuate pro-inflammatory signaling and facilitate a pro-resolving environment by inducing COX1–mediated PGJ₂ production [100]. Upon Epo stimulation, it is reported that elevated PGJ₂ inhibits Wnt signaling to promote the transition of SEP from proliferation to differentiation [55]. Whether selenium and selenoproteins serve as potential key regulatory factors of stress erythroid progenitor development within the EBI through this pathway is not well understood and is currently being investigated. In addition, the effect of dietary selenium on steady state erythropoiesis also needs to be further studied. Despite broader expression of selenoproteins in erythroblasts, only a few selenoenzymes (e.g., GPX1 and GPX4) have been studied in detail for their roles in ferroptosis and detoxification of H_2_O_2_ and lipid hydroperoxides. The impairment of stress erythropoiesis in Se-D mice suggests potential functions for selenium-sensitive selenoproteins, including SELENOW, SELENOP, and SELENOH, in redox regulation under stress. Moreover, because ER stress is pivotal in erythroid differentiation and inflammation-induced anemia, ER-resident selenoproteins such as SELENON, SELENOO, and SELENOS may also contribute through calcium homeostasis and proteostasis. Future studies dissecting individual selenoprotein functions may reveal new therapeutic targets for anemia and inflammation-associated hematopoietic disorders.

## Data Availability

No datasets were generated or analysed during the current study.
